# An Unusual Case of Multifactorial Catatonia Following Taxane‐Based Chemotherapy Regimen

**DOI:** 10.1155/crps/5820055

**Published:** 2026-04-16

**Authors:** Wei Siang Lee, Leslie Lim, Vincent Wong

**Affiliations:** ^1^ Department of Psychiatry, Singapore General Hospital, Singapore, Singapore, sgh.com.sg

## Abstract

Catatonia is a neuropsychiatric syndrome characterised by motor and behavioural disturbances. While commonly associated with psychiatric conditions, a substantial proportion of cases have medical or drug‐induced aetiologies. Chemotherapy can cause catatonia, and taxane‐based chemotherapy is known to cause central neurotoxicity; however, taxane‐related catatonia has not been reported before. We describe a 75‐year‐old woman with a schizophrenia in remission, who developed catatonia after docetaxel administration for breast cancer. Her catatonia fulfilled DSM‐5‐TR criteria and had an initial Bush–Francis catatonia rating scale (BFCRS) score of 23. A lorazepam challenge was positive, and treatment with oral lorazepam resulted in rapid resolution. Mild recurrence during follow‐up was successfully managed with lorazepam titration. We discuss the possible link between taxanes, taxane‐induced central neurotoxicity (TICN) and catatonia, with neuroscientific, clinical and radiological considerations. This case highlights the potential contribution of taxane chemotherapy to catatonia in a medically complex patient, and underscores the importance of early recognition and prompt treatment of catatonia.

## 1. Introduction

Catatonia is a complex neuropsychiatric syndrome characterised by abnormalities of movement and behaviour. It is heterogenous in its clinical presentation, which ranges from stuporous and withdrawn, to hyperkinetic and agitated with combativeness [1]. Catatonia can result in serious morbidity, including dehydration and malnutrition, haemodynamic instability, complications of immobility and injury to self and others. Therefore, it is important for clinicians to recognise and treat catatonia promptly, including addressing any causative medical or psychiatric conditions.

Often, catatonia is described in association with psychiatric conditions, including major depressive disorder, bipolar disorder, psychotic disorders and autism spectrum disorder. Indeed, the DSM‐5‐TR includes a catatonia specifier for the aforementioned conditions [2].

Nonetheless, a significant proportion of catatonia cases are associated with a medical aetiology [3]. Revealingly, a systematic review investigated how often catatonia in hospital patients was due to a medical aetiology; this was found to be 20% in the general hospital population, but rose to 50% in the acute medical and surgical settings, and more than 80% among elderly adults seen by consult psychiatry [4].

These myriad medical aetiologies include neurological, endocrine, autoimmune, infectious and metabolic diseases. Chemotherapy and immunosuppressive agents have also been linked to catatonia. For example, a pharmacovigilance study of the FDA adverse event reporting database found 66 reports of tacrolimus‐related catatonia, with an elevated reporting odds ratio within those older than 40 years [5].

Despite this, there are hitherto no reports or studies linking catatonia with taxane‐based chemotherapy. This may be surprising to physicians familiar with the ‘chemobrain’, or taxane‐induced central neurotoxicity (TICN), that some breast cancer patients experience after taxane administration.

In this report, we present a general hospital inpatient whose catatonia emerged after receiving taxane‐based chemotherapy, namely docetaxel, and having also suffered acute kidney injury and delirium.

## 2. Case Presentation

A 75‐year‐old married housewife received taxane‐based chemotherapy for breast cancer, and was subsequently admitted to a tertiary general hospital in Singapore for medical delirium, followed by catatonia. Her prior medical history is significant for diabetes mellitus, hypertension and a right lentiform nucleus infarct 7 years prior.

Her prior psychiatric history is significant for schizophrenia. This was first diagnosed 30 years ago (aged 45) with auditory hallucinations, passivity phenomena, grandiose delusions and delusions of pregnancy. She had two previous admissions for psychotic episodes, once 30 years ago, and again 7 years ago. In the community, she was kept well on risperidone 2 mg/day.

1.5 months before her catatonia, she was diagnosed with stage 3 invasive lobular carcinoma of the breast. She consented to receiving neoadjuvant chemotherapy. On cycle 1 day 1, she received intravenous docetaxel 86 mg (at 1.8 mg/kg) and subcutaneous Phesgo (pertuzumab 1200 mg and trastuzumab 600 mg). One day later, she received subcutaneous pegfilgrastim (granulocyte colony stimulating factor) 6 mg.

Two days after initiating chemotherapy, she developed nausea, vomiting, poor oral intake and lethargy. This lasted 3 weeks before she finally presented to the emergency department. She was found to have severe acute kidney injury (serum creatinine 1077 mmol/L), metabolic acidosis, leucocytosis possibly from septic aetiology and hypoactive delirium. Computed tomography of the brain did not show any intracranial metastasis. She was, therefore, admitted under the hospital’s oncology service for medical treatment. She received IV piperacillin–tazobactam, IV crystalloid hydration and nasogastric feeding.

Due to her severe renal impairment, her risperidone was initially withheld on admission. On the 14^th^ day of admission, she was reviewed by the psychiatry consultation–liaison service, found to be drowsy and inattentive and assessed to be in delirium, but not in relapse of schizophrenia. On the 19^th^ day of admission, she remained in delirium, but began to manifest shouting behaviour as well. Therefore, her risperidone was re‐initiated at 0.5 mg/day.

On the 21^st^ day of admission, she developed catatonia fulfilling DSM‐5‐TR criteria, with features of stupor, staring, echolalia, stereotypy, verbigeration, waxy flexibility and perseveration. She had an initial Bush–Francis catatonia rating scale (BFCRS) score of 23, and a positive response to an oral lorazepam 0.5 mg challenge. There were no psychotic symptoms, and no relapse signature relevant to her schizophrenia.

Her catatonia was treated with oral lorazepam 1.5 mg/day and risperidone 1 mg/day, with good clinical response. By the 29^th^ day of admission, her catatonia had resolved, with a BFRCS score of 2. Her medical issues had also resolved, and she was discharged home with PO lorazepam 0.75 mg/day and risperidone 1 mg/day.

Over 2 months of follow‐up in the psychiatry clinic, her BFCRS transiently rose to 9, even though there was no longer any AKI or delirium present. In response, we up‐titrated her PO lorazepam to 3 mg/day, and her BFCRS reduced to 2, representing a remission of catatonia. Throughout this period, she showed satisfactory oral intake and stable vital signs, and she was treated as an outpatient without any further complications. She also remained in remission from her schizophrenia. Her family expressed satisfaction with her psychiatric outcomes.

## 3. Discussion

Having presented a case of catatonia following taxane administration, we now present the plausibility of an association in neuroscientific, clinical and radiological terms.

Taxanes are anti‐neoplastic agents used in treating cancers of the breast, lung, gastrointestinal, prostate and bladder, among others. Docetaxel and paclitaxel are the most commonly used taxanes. They work by binding to polymerised tubulins, thereby stabilising microtubules and interrupting the G2 phase of the cell cycle. They also bind to mitochondrial tubulin, damaging mitochondria and triggering cell apoptosis [6].

Taxanes are known to cause neurotoxicity in the central nervous system (CNS). This TICN can present as encephalopathy [7], ataxia, emotional distress or cognitive impairment. The risk of cognitive impairment is further elevated with older age and higher doses of docetaxel received. Although the underlying mechanisms of TICN are poorly understood, it has been postulated that because taxanes interfere with microtubule function, they affect synaptic plasticity and memory formation; additionally, increased circulating levels of pro‐inflammatory cytokines after taxane administration can mediate neuroinflammation, apoptosis and manifest with cognitive disturbances [6].

In breast cancer survivors who had received chemotherapy (not limited to taxanes), various neuroimaging abnormalities have been described. The most consistent structural MRI findings are grey matter volume reductions in the frontal and temporal lobe regions, including the hippocampus; these were related to chemotherapy induced cognitive impairment. This acute structural damage may partially recover after 1 year. Reduced cerebral blood flow in bilateral frontal and parietal lobes has also been described [8]. While the structural neuroimaging findings in catatonia are more varied, it similarly features frontal lobe abnormalities, along with basal ganglia and diffuse white matter abnormalities. Functional imaging in catatonia has also demonstrated hypometabolism in the frontal and temporal lobes [9].

Even though taxane‐related catatonia has hitherto not been described, it is clear that taxanes exert neurotoxic effects on the CNS; furthermore, structural and perfusion abnormalities of the frontal lobes occur both after chemotherapy administration, and in catatonia.

Therefore, while our patient’s catatonia is likely multifactorial in aetiology, with dehydration, acute kidney injury and acidosis the probable culprits, we postulate that docetaxel (and perhaps the co‐administration of pertuzumab and trastuzumab) significantly contributed to her catatonia. This is especially so considering that her catatonia recurred despite the resolution of her medical conditions.

Whether treatment with risperidone was appropriate may be a subject of debate. While some authors advise against treatment of catatonia with antipsychotics [10], there is also evidence of antipsychotic use, including trials of risperidone yielding benefit in catatonia [11, 12]. Furthermore, we must also consider that her catatonia occurred following the withdrawal of risperidone, which had kept her schizophrenia in remission for years.

Notwithstanding the pathophysiology of catatonia, this case demonstrates good clinical response to a benzodiazepine.

## Funding

No funding was received for this manuscript.

## Disclosure

This case has previously been published as an abstract in the BJPsych Open [13].

## Consent

The patient has consented to this case being published as a case report.

## Conflicts of Interest

The authors declare no conflicts of interest.

## Data Availability

The data that support the findings of this study are available from the corresponding author upon reasonable request.
